# Astragaloside IV alleviates mouse slow transit constipation by modulating gut microbiota profile and promoting butyric acid generation

**DOI:** 10.1111/jcmm.15586

**Published:** 2020-07-06

**Authors:** Qiulan He, Changpeng Han, Liang Huang, Haojie Yang, Jiancong Hu, Huaxian Chen, Ruoxu Dou, Donglin Ren, Hongcheng Lin

**Affiliations:** ^1^ Department of Anaesthesiology First Affiliated Hospital of Sun Yat‐sen University Guangzhou China; ^2^ Department of Colo‐proctology Yueyang Hospital of Integrated Traditional Chinese and Western Medicine Shanghai University of Traditional Chinese Medicine Shanghai China; ^3^ Department of Colorectal Surgery The Sixth Affiliated Hospital of Sun Yat‐sen University Guangzhou China; ^4^ Guangdong Institute of Gastroenterology The Sixth Affiliated Hospital of Sun Yat‐sen University Guangzhou China; ^5^ Guangdong Provincial Key Laboratory of Colorectal and Pelvic Floor Diseases The Sixth Affiliated Hospital of Sun Yat‐sen University Guangzhou China

**Keywords:** Astragaloside IV, butyric acid, gut microbiota, short‐chain fatty acid, slow transit constipation

## Abstract

Gut microbiota and short‐chain fatty acids (SCFAs) are associated with the development of various human diseases. In this study, we examined the role of astragaloside IV in modulating mouse gut microbiota structure and the generation of SCFAs, as well as in slow transit constipation (STC). An STC model was established by treating mice with loperamide, in which the therapeutic effects of astragaloside IV were evaluated. The microbiota community structure and SCFA content were analysed by 16S rRNA gene sequencing and gas chromatography‐mass spectrometry, respectively. The influence of butyrate on STC was assessed using a mouse model and Cajal cells (ICC). Astragaloside IV promoted defecation, improved intestinal mobility, suppressed ICC loss and alleviated colonic lesions in STC mice. Alterations in gut microbiota community structure in STC mice, such as decreased *Lactobacillus reuteri* diversity, were improved following astragaloside IV treatment. Moreover, astragaloside IV up‐regulated butyric acid and valeric acid, but decreased isovaleric acid, in STC mouse stools. Butyrate promoted defecation, improved intestinal mobility, and enhanced ICC proliferation by regulating the AKT–NF‐κB signalling pathway. Astragaloside IV promoted intestinal transit in STC mice and inhibited ICC loss by regulating the gut microbiota community structure and generating butyric acid.

## 1 INTRODUCTION

Chronic constipation is a common intestinal disease characterized by difficulty in the passage of stools or infrequent bowel movements, which usually associated with significant utilization of healthcare resources .^1^, ^2^ Its global prevalence is reported to be 14%,^3^ and it has been identified as a risk factor for some gastrointestinal disorders including colorectal neoplasms.^4^ Slow transit constipation (STC) is a major type of chronic constipation characterized by a substantial increase in bowel transit time, but with no alteration in bowel diameter and the absence of megacolon and aganglionosis.^2^ Regarding the current clinical management of STC, this mainly relies on the use of symptomatic treatments, such as wetting agents and osmotic laxatives. However, these treatments are of limited efficacy because of their adverse effects and refractoriness, eventually necessitating surgical operations, such as partial or total colectomy.^2^, ^5^ A decreased volume of interstitial cells of Cajal (ICCs), which play a key role in regulating intestinal motility, is believed to be involved in the pathophysiology of STC.^6^ Moreover, the reduction of ICC in STC patients has been shown to be associated with the suppression of c‐Kit expression and its interaction with ligand stem cell factor, which controls the development of ICCs and their survival in the intestines.^7^ In our previous study, it was also found that ICC number in the rectum with chronic constipation decreased significantly compared with those in normal patients.^8^ However, the pathogenesis of STC and the molecular mechanisms underlying it remain poorly understood.

Over the past decade, increasing attention has been focused on the gut microbiome and host‐microbe interactions because of increasing evidence of their essential physiological and pathogenic roles, which have come to light following technical developments in culture‐independent analyses.^9^, ^10^ The human gut microbiome is composed of several different types of microbial species, including bacteria, archaea, eukaryotic microbes and viruses.^9^ Through modulating metabolism, pathogen invasion and the immune system, the gut microbiota has been widely implicated in the pathogenesis of various human disorders, such as irritable bowel syndrome (IBS), *Clostridium difficile* infection, cardiovascular disease and inflammatory bowel disease (IBD). Significant differences in the composition of the gut microbiota have recently been identified between the two common gastrointestinal diseases IBS and IBD, which have been used to distinguish patients suffering from these two conditions.^11^ Changes in the composition of the gut microbiota have also been reported to be involved in the pathogenesis of constipation. A recent 16S rRNA‐based microbial profiling analysis demonstrated remarkable depletions of *Roseburia*, *Coprococcus* and *Bacteroides* in stool samples from patients with functional constipation, which were linked to alterations in carbohydrate, fatty acid, and lipid metabolism.^12^ These discoveries indicate that the regulation of gut microbiota profiles and short‐chain fatty acid (SCFA) generation could be promising targets for the development of new anti‐STC drugs.^12^

*Astragalus membranaceus* (AM) is a medicinal herb widely used in traditional Chinese medicine. Its main pharmacological action is ‘Yi qi gu biao’. ‘*Jin gui yi*’ first proposed that AM could be used to treat chronic constipation and that a clinical prescription containing astragalus could significantly improve constipation. Astragaloside IV (As‐IV) is a major bioactive triterpenoid chemical present in AM.^13^, ^14^ Previous pharmacological studies showed that it is beneficial in a number of human disorders, such as breast cancer,^14^ renal interstitial fibrosis,^14^ acute kidney injury and inflammation,^15^ and focal cerebral ischemia.^16^ Moreover, autophagy and oxidative stress in mouse intestines caused by intestinal microbiota during the onset of acute ischemic stroke were also shown to be effectively reversed by As‐IV.^17^ The disposition of As‐IV through the enterohepatic circulation and its therapeutic effects has been shown to be modulated by the intestinal microbiota.^18^ These reports identify a close interactive modulation between As‐IV and the intestinal microbiome. However, the influence of As‐IV on the gut microbiome in the context of constipation remains unknown.

In the present study, we investigated the anti‐constipation effects of As‐IV using a loperamide‐induced STC mouse model, followed by 16S rRNA microbial profiling analysis and SCFA quantification to identify the mechanisms of STC inhibition induced by As‐IV, and direct clinical treatments of chronic constipation.

## 2 MATERIALS AND METHODS

### 2.1 Animals and grouping

Kunming (KM) mice with a bodyweight of 35.0 ± 2.0 g were obtained from the Guangdong Medical Experimental Animal Center (Guangdong, China) and adapted to the standard laboratory environment (ambient temperature: 23 ± 1°C; light–dark cycle: 12/12 hours; relative humidity: 45%–65%) for 1 week with food and drinking water provided ad libitum. All animal experiments in this study were approved by the Animal Care Review Committee of Sun Yat‐sen University and carried out strictly in accordance with the Guidelines for the Care and Use of Laboratory Animals (National Institutes of Health, USA).

For microbiota profiling, a total of 30 male and 30 female mice were randomly divided into five groups (six male and six female mice in each group): control group, STC (model) group, STC‐LD group (STC model treated with low‐dosage, 10 mg/kg As‐IV), STC‐MD group (STC model treated with median‐dosage, 30 mg/kg As‐IV), and STC‐HD group (STC model treated with high‐dosage, 90 mg/kg As‐IV). For the experiment involving treatment with sodium butyrate, 24 mice were randomly divided into three groups (four male and four female mice in each group): control group, STC group and STC + butyrate group.

### 2.2 STC model establishment and treatments

**Figure 1:**
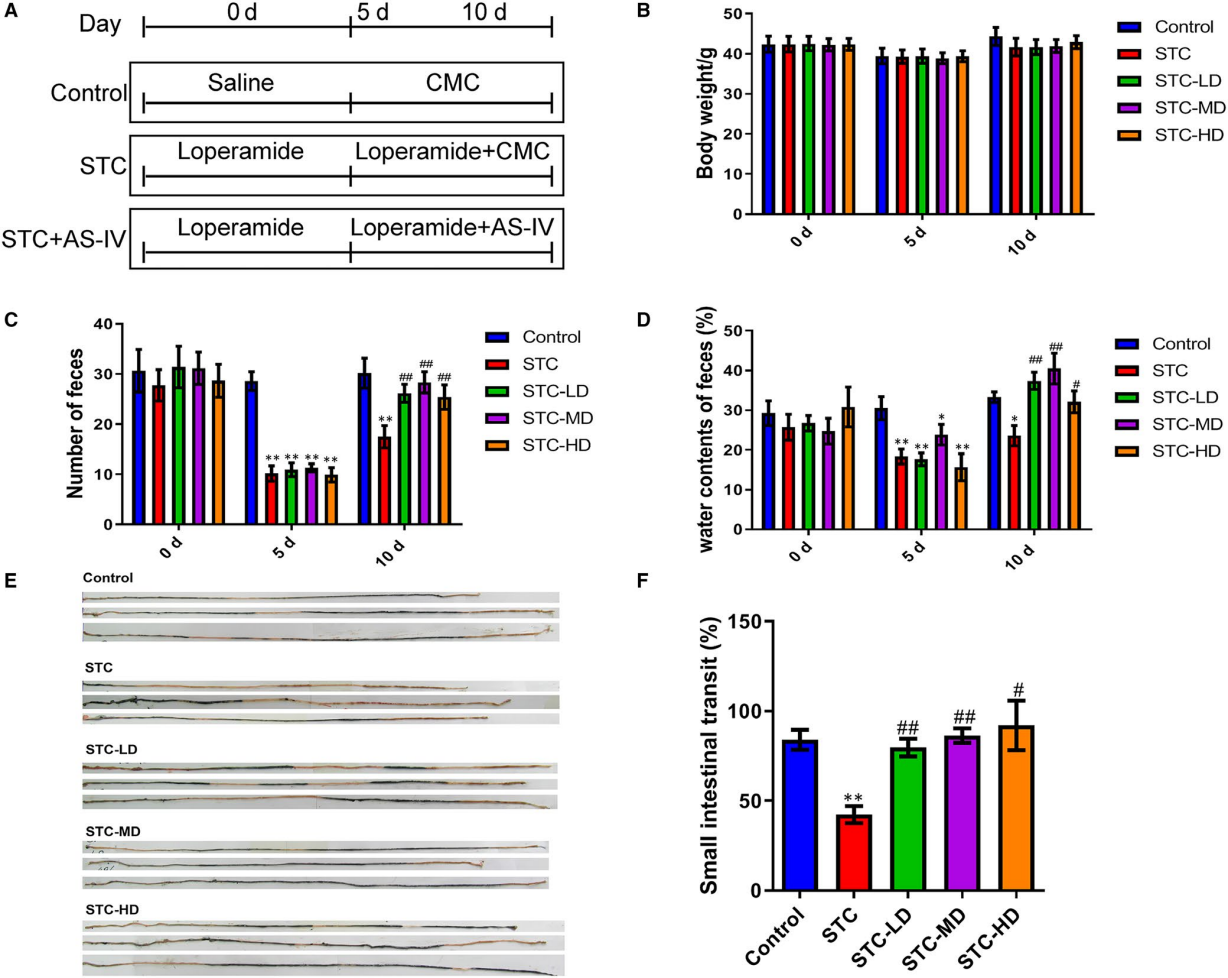
Improved defecation and intestinal transit in loperamide‐induced STC mice by astragaloside IV. A, Schematic presentation of STC model establishment and As‐IV treatment. Sixty mice were divided into control, STC, STC‐LD, STC‐MD and STC‐HD groups. The STC‐LD, STC‐MD and STC‐HD groups were treated with 10 mg/kg, 30 mg/kg and 90 mg/kg As‐IV, respectively, for 5 d. B‐D, Alterations of bodyweights (B), number of faeces (C) and faecal water content (D) in STC model mice treated with As‐IV. Mouse bodyweight, faeces number and faecal water content were determined at days 0, 5 and 10 during the experiment. E, F, Influences of treatment with As‐IV on the small intestinal transit in loperamide‐induce STC mice. Intestinal transit was assessed by the intestinal propelling movement of carbon ink (E) and quantitatively compared among the control, STC, STC‐LD, STC‐MD and STC‐HD groups (F). As‐IV: astragaloside IV; STC: slow transit constipation; CMC: carboxymethyl cellulose sodium; LD: low dosage; MD: median dosage; HD: high dosage; *P < .05, **P < .01 (comparison with control group); ^#^ *P *< .05, ^##^ *P* < .01 (comparison with STC group)

The loperamide‐induced mouse STC model was established as previously described, with minor modifications.^19^ After acclimation to the conditions for 1 week, mice in the STC, STC‐LD, STC‐MD and STC‐HD groups were treated with loperamide (10.0 mg/kg bodyweight) by oral gavage twice a day for 10 days. Starting on the sixth day, the STC group was co‐treated with carboxymethyl cellulose sodium (CMC) during loperamide administration for 5 days, and the STC‐LD, STC‐MD and STC‐HD groups were given an As‐IV solution soluble in CMC with different dosage (10 mg/kg, 30 mg/kg and 90 mg/kg, respectively) by oral gavage 1 hours after each loperamide administration for 5 days. Mice in the control group were treated with the same volume of saline solution by gavage for 5 days, followed by oral gavage administration of CMC for another 5 days (see Figure 1A). The STC + butyrate group was given 1.1% sodium butyrate in the drinking water. The defecation frequency, wet stool weight and dry stool weight were recorded each day. On the 11th day, all mice were sacrificed after stool collection and mouse serum and proximal colonic tissues (10 mm) were collected for further analysis.

### 2.3 Faecal water content and colonic motility

In this study, the water content of mouse faeces was calculated as previously described.^19^ The mouse colonic motility was evaluated by the intestinal propelling movement of carbon ink, which was quantified as the small intestinal transit (%). Three mice in each group were treated with 0.2 g/mL carbon powder (20 mL/kg bodyweight) on the 10th day during the model establishment. Mice were sacrificed 30 minutes later and the small intestines between the pylorus and ileocecal region were collected and immediately extended in order to calculate the small intestinal transit.^20^ Following this, proximal colonic tissues were rinsed with 30 mm of normal saline solution and stored at −80°C for further analysis.

### 2.4 Histopathological examination

Histological examination of colonic tissues was carried out as previously described.^20^ For haematoxylin and eosin staining, mouse colonic tissues were fixed with 10% buffered formalin at room temperature, dehydrated with graded doses of alcohol, embedded in paraffin, and sliced into 5‐µm‐thick sections. Colonic tissue slices were deparaffinized and stained with haematoxylin and eosin, mounted with neutral balsam, and subjected to analyses of histological morphology, mucosal thickness and muscle thickness.

### 2.5 Immunohistochemistry

The distribution of ICCs and expression of c‐Kit in colonic tissues were analysed by immunohistochemistry. Tissue slides were dewaxed with xylene, rehydrated, heated in a citrate buffer for antigen retrieval, incubated in 3% H_2_O_2_ for 5 minutes in order to quench endogenous peroxidase, blocked with normal goat serum for 15 minutes, incubated with anti‐c‐Kit antibodies (Abcam; 1:200) for 2 hours at 37°C, washed with PBS, incubated with secondary antibodies for 15 minutes at 37°C, and finally developed with the DAB horseradish peroxidase colour development kit (GBCBIO Technologies, Guangzhou, China). After being counterstained with haematoxylin, slides were mounted with neutral balsam and observed with a light microscope. Additionally, images were analysed using Image Pro Plus software (Media Cybernetics Inc).

### 2.6 16S rRNA microbial profiling analysis

The community structures of mouse gut microbiota were analysed with a 16S rRNA gene sequencing technique, as previously described with minor modifications.^21^ Total DNA samples were extracted from mouse stools using the FastDNA Spin Kit for Soil (#6560‐200; MP Biomedicals), in line with the manufacturer's instructions, and were subjected to PCR amplification using primers targeting the V3 and V4 regions of the 16S rRNA sequences. The PCR products were subjected to the establishment of a sequencing library using the TruSeq DNA LT sample preparation kit (Illumina, USA) and sequenced using the Illumina Hiseq 2500 platform. Raw data from sequencing were analysed using Quantitative Insights into Microbial Ecology (QIIME) 1.5.0. After the screening of raw sequences and removal of short sequences (<200 bp), sequences with a similarity of >97% were clustered into the same operational taxonomic unit (OTU) using the open‐reference OTU picking method, followed by comparison based on OTU homology and classification based on species. Rarefaction curves were constructed based on Chao1 and Observed Species indexes. The bacterial diversity within the samples (alpha diversity) was evaluated based on Chao1 and Observed Species indexes using QIIME. The bacterial diversity between samples (beta diversity) was analysed based on an Unweighted UniFrac index using principal coordinates analysis (PCoA) plots. The relative abundances at phylum, order and genus levels between the different groups were compared separately, based on species with a relative abundance of >1%.

### 2.7 SCFA quantification

The SCFA content in mouse stool samples was measured using gas chromatography‐mass spectrometry (GC/MS). Stool samples of 100 mg were homogenized with 100 μL of 15% phosphoric acid, 100 μL of 250 μg/mL isocaproic acid, and 400 μL of diethyl ether, and centrifuged at 12 000 rpm for 10 minutes. The supernatants (sample volume: 1 μL) were analysed using the Agilent 6890N/5975B GC/MS machine equipped with an Agilent HP‐INNOWAX column (30 m * 0.25 mm ID * 0.25 μm) under full wave massing scan. Helium (flow rate: 1.0 mL/min, split ratio 10:1) was used as the carrier gas. The injection and ionization temperatures were 240°C and 230°C, respectively. The SCFA concentrations were calculated according to standard curves established using acetic acid, propionic acid, butyric acid, isovaleric acid, isobutyric acid, valeric acid and caproic acid.

### 2.8 ICC cell isolation and treatment

Newborn mice (2‐6 days) were fasted for 12 hours and sacrificed for the collection of colonic tissues, which were sliced into 0.5 mm × 0.5 mm pieces and maintained in 24‐well plates filled with RPMI1640 medium (Gibco) containing 10% foetal bovine serum and 1% penicillin and streptomycin at 37°C in a culture chamber with 5% CO_2_. The culture medium was replaced every 3‐4 days, and the growth of mouse ICCs was observed daily. The experiment of cell isolation was repeated three times.

When cell confluence of over 70% was reached, the identity of the ICCs was verified using the expression of c‐Kit and vimentin (#ab92547; Abcam) proteins measured by immunofluorescence, as previously described.^22^ ICC cells were treated with 0, 0.00005, 0.0005, 0.005, 0.05 and 0.5 mmol/L sodium butyrate for 4 hours.

### 2.9 Western blotting

Total proteins were extracted from mouse tissues or cells using RIPA Lysis Buffer (#P0013B; Beyotime), following the manufacturer's instructions. A total of 30 μg of protein from each sample were boiled at 100°C for 5 minutes, separated through SDS‐PAGE, and transferred onto PVDF membranes (Millipore). The membranes were blocked with 5% lipid‐free milk for 1‐2 hours, incubated with primary antibodies diluted in TBST for 2 hours at room temperature, washed with TBST and incubated with secondary antibodies for 1 hour at room temperature. Protein abundances were determined by development with enhanced chemiluminescence (ECL) substrates (Millipore). GAPDH was used as an internal standard. Primary antibodies targeting c‐Kit (#ab256345; Abcam), P65 (#ab16502; Abcam), p‐P65 (#ab86299; Abcam), AKT (#9272; CST), p‐AKT (#4060; CST) and GAPDH (#ab8245; Abcam) were applied.

### 2.10 Statistical analysis

Quantitative data in this study are presented as mean ± standard deviation (SD) and were analysed using SPSS 20.0 software. Differences between groups were evaluated using analysis of variance. Correlations were assessed using the Pearson method. Significance was defined by a *P* value of <.05.

## 3 RESULTS

### 3.1 Astragaloside IV promotes defecation and intestinal mobility in loperamide‐induced STC mice

To investigate the influence of As‐IV on STC, we established a mouse STC model via oral gavage administration of loperamide and applied different dosages of As‐IV (Figure 1A). Mice in the STC‐LD, STC‐MD and STC‐HD groups were given an As‐IV solution with different dosage (10 mg/kg, 30 mg/kg and 90 mg/kg, respectively). The bodyweights of the five groups showed no significant differences after model establishment and As‐IV treatment (Figure 1B). However, both the number of faeces and faecal water content of the STC group were significantly decreased at day 5 during model establishment, compared with those in the control group (Figure 1C,D). At day 10, faeces number and faecal water content were markedly recovered following As‐IV treatment in the STC‐LD, STC‐MD and STC‐HD groups, but not in the STC group (Figure 1C,D). The small intestinal transit in the STC group was remarkably lower than that in the control group, as shown by the intestinal propelling movement of carbon ink (Figure 1E,F). However, the STC‐LD, STC‐MD and STC‐HD groups showed greatly recovered small intestinal transit, in comparison with the STC group (Figure 1E,F). These results indicate that the treatment with As‐IV effectively promoted defecation and enhanced colonic mobility in mice with loperamide‐induced STC.

### 3.2 Astragaloside IV recovered Cajal cell number and alleviated colonic lesion in loperamide‐induced STC mice

**Figure 2:**
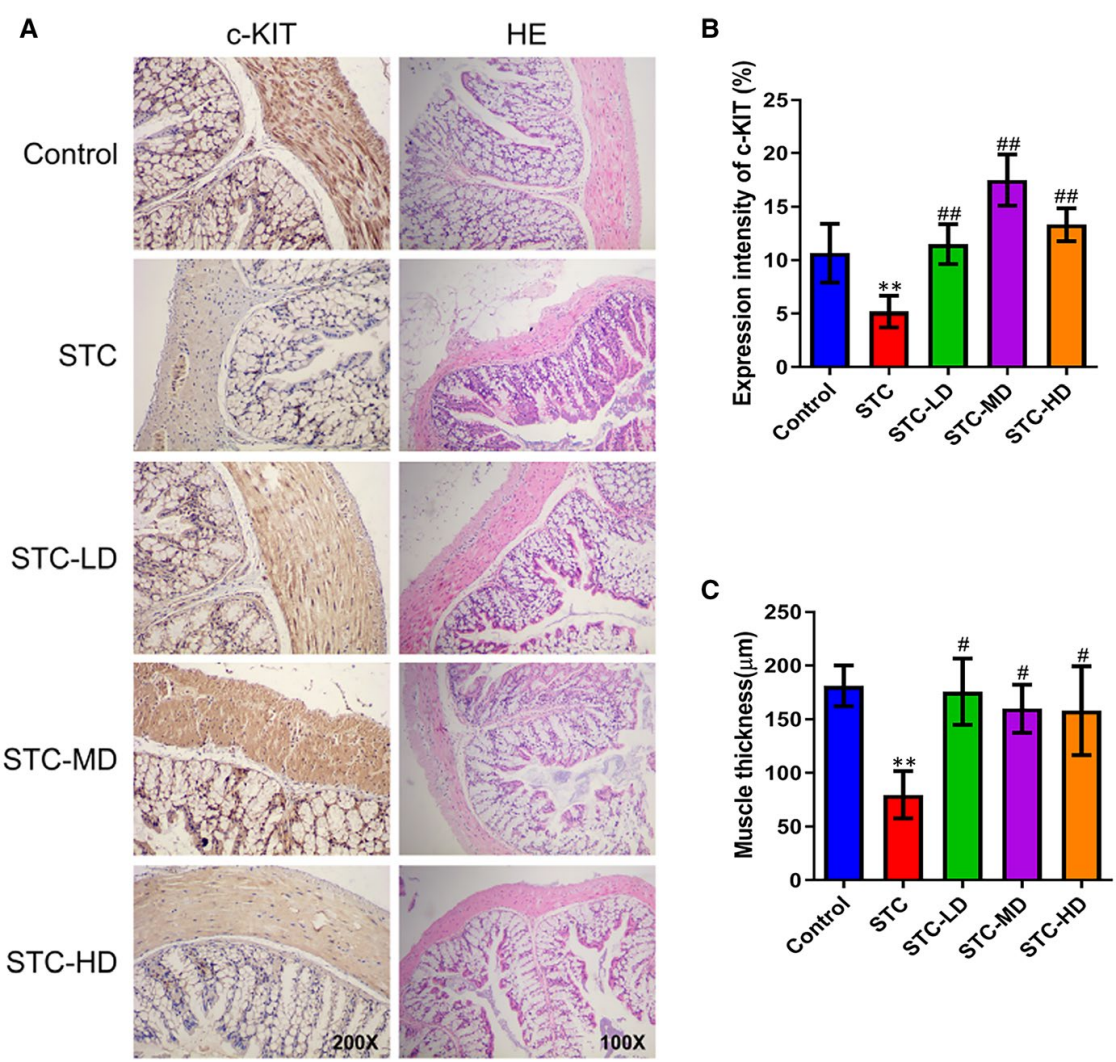
Inhibition of ICC loss and intestinal lesions by astragaloside IV in loperamide‐induced STC mice. A, ICC distribution and pathogenic alterations in colonic tissues of STC mice treated with As‐IV at different dosages. Immunohistochemistry targeting c‐Kit (left) (magnification, 200×) and H&E staining (magnification, 100×) were performed for pathogenic evaluation. Mice in the STC‐LD, STC‐MD and STC‐HD groups were treated with 10 mg/kg, 30 mg/kg and 90 mg/kg As‐IV, respectively. B, Quantitation of c‐Kit expression levels in colonic tissues of STC mice treated with As‐IV. C, Alteration of muscle thickness in the colonic tissues of STC mice after treatment with As‐IV. As‐IV, astragaloside IV; STC, slow transit constipation; LD, low dosage; MD, median dosage; HD, high dosage; HE, haematoxylin and eosin; **P < .01 (comparison with control group); ^#^ *P* < .05, ^##^ *P* < .01 (comparison with STC group)

Through immunohistochemistry combined with H&E staining, we found that ICCs were distributed in both the muscular and the submucosa layers of the mouse colonic tissues, but that ICC numbers in the STC group were remarkably lower than in the control group (Figure 2A). Treatments with low‐, median‐ or high‐dosage As‐IV caused significant increases in ICC numbers in the STC group (Figure 2A). We observed that the expression levels of c‐Kit in the colonic tissues of the STC group were markedly down‐regulated compared with those in the control group and were then elevated following treatment with As‐IV (Figure 2B). The median dosage resulted in the greatest elevation of c‐Kit expression in colonic tissues of STC mice (Figure 2B). Pathogenic evaluation also showed that, compared with the control group, the STC group had significant colonic lesions and showed the loss of goblet cells in mucosa, lymphocyte infiltration, myenteric plexus dysregulation, decrease in muscle thickness, and obvious oedema and hyperaemia in the longitudinal muscle layer (Figure 2A,C). However, the pathogenic alterations in colonic tissues of STC mice were significantly alleviated by treatments with As‐IV at low, median and high dosages (Figure 2A,C).

### 3.3 Astragaloside IV altered gut microbiota diversity in loperamide‐induced STC mice

**Figure 3:**
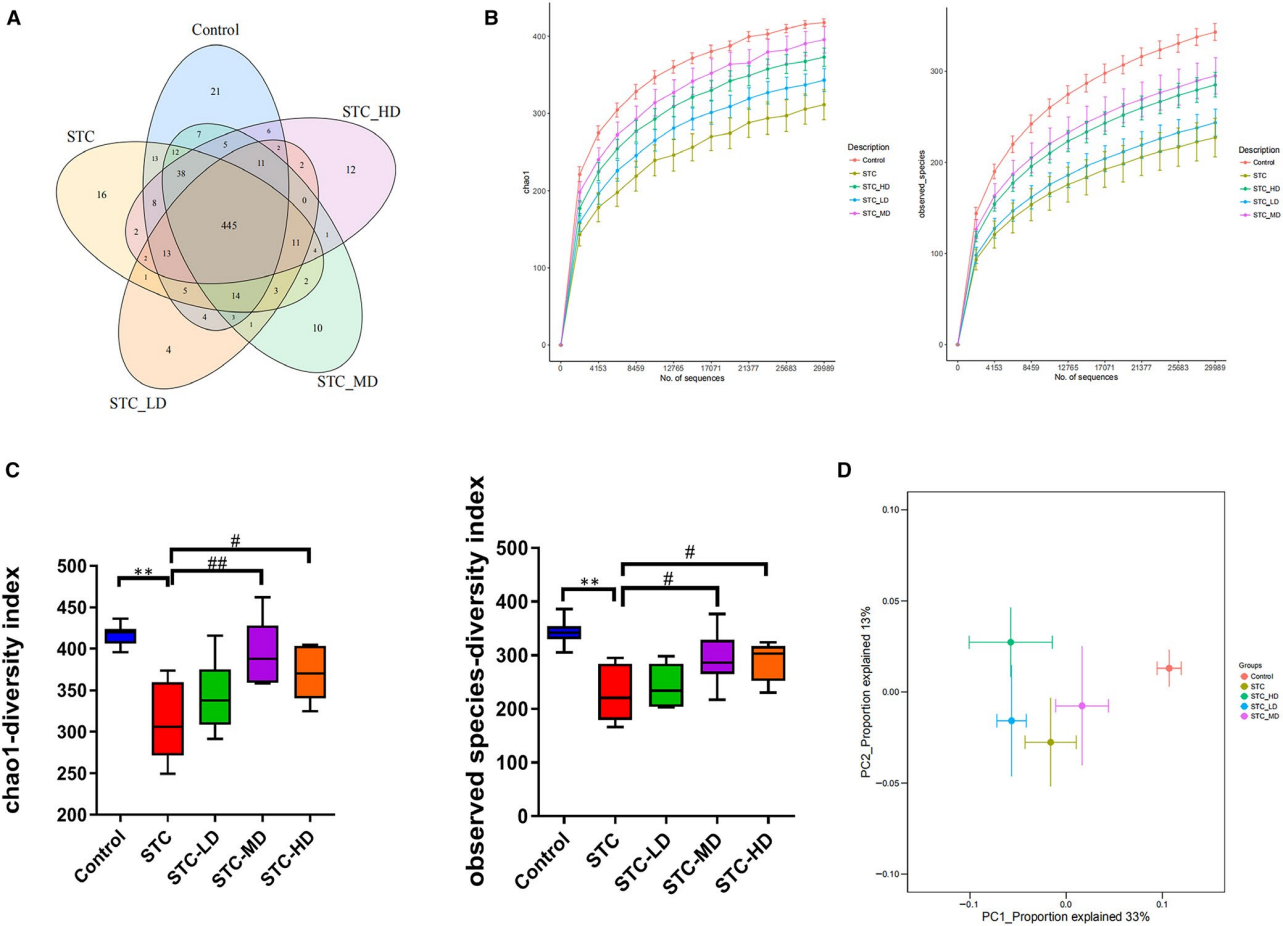
Regulation of gut microbiota community structure by astragaloside IV in loperamide‐induced STC mice. A, A Venn diagram representing the OTU numbers identified in the gut microbiome of STC mice treated with As‐IV; As‐IV at different dosages. Mice treated with 10 mg/kg, 30 mg/kg and 90 mg/kg As‐IV were designated as the STC‐LD, STC‐MD and STC‐HD groups, respectively, and were subjected to 16S rRNA microbial gene sequencing. B, The rarefaction curves established based on the Chao1 (left) and Observed Species (right) indexes of the 16S rRNA microbial profiling analysis. C, The alpha diversity of microbiomes identified in STC mice treated with As‐IV. The microbial abundances within the group were compared based on the Chao1 and Observed Species separately. D, The beta diversity of microbiomes characterized in the STC model mice that underwent As‐IV treatment. Microbiome diversity between the groups was assessed through PCoA analysis based on Unweighted UniFrac. As‐IV, astragaloside IV; STC, slow transit constipation; LD, low dosage; MD, median dosage; HD, high dosage; PCoA, principal coordinates analysis; **P < .01 (comparison with control group); ^#^ *P* < .05, ^##^ *P* < .01 (comparison with STC group)

To explore the potential involvement of the gut microbiome in the effects of the treatment of STC with As‐IV, we analysed the community structures of gut microbiota in loperamide‐induced STC mice treated with As‐IV using 16S rRNA microbial profiling analysis. A total of 1120 OTUs were identified in the above‐mentioned five groups, including 607 OTUs in the control group, 589 OTUs in the STC group, 521 OTUs in the STC‐LD group, 567 OTUs in the STC‐MD group and 562 OTUs in the STC‐HD group (Figure 3A). Among these, 548 OTUs were identified in both the control and the STC groups, and 497, 535 and 528 OTUs in the control group were also identified in the STC‐LD, STC‐MD and STC‐HD groups, respectively (Figure 3A). The rarefaction curves flattened out in correlation with the increases of Chao1 and Observed Species indexes, indicating the preferable sequencing depth and high coverage of species (Figure 3B). Alpha diversity analysis showed that microbial abundances in the STC group were significantly lower than in the control group and were remarkably increased by median‐ or high‐dosage As‐IV treatment (Figure 3C). Median‐dosage As‐IV induced the greatest increase of microbial abundance in loperamide‐induced STC mice (Figure 3C). Through beta diversity analysis using PCoA based on the Unweighted UniFrac index, we showed that the control group was closer to STC‐MD, other than the STC group, indicating the high similarity in microbiota community structure between the STC‐MD and control groups (Figure 3D).

**Figure 4:**
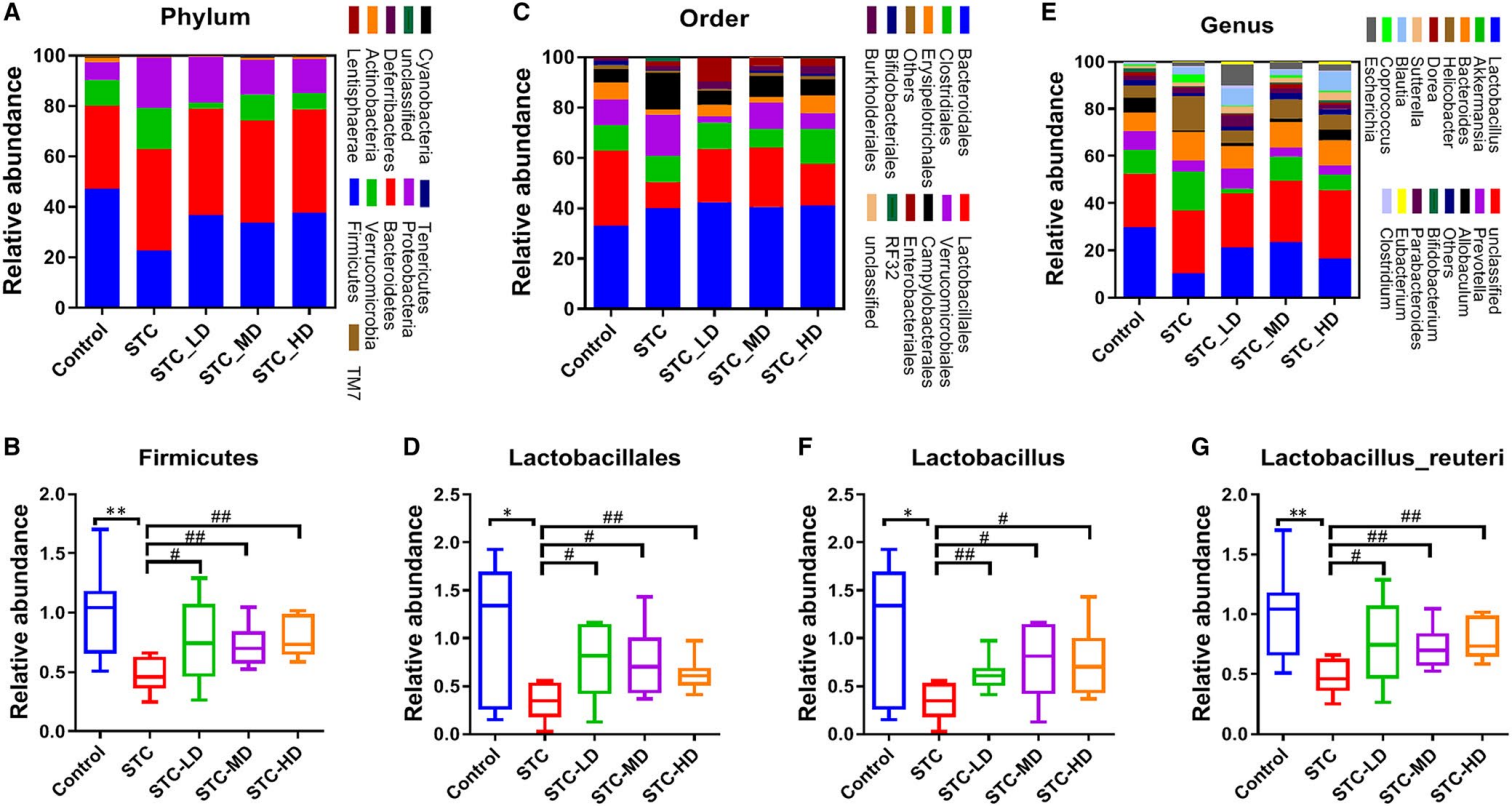
Microbiome alterations at distinct taxonomic levels in STC mice caused by astragaloside IV treatment. Relative abundances of microbiota at three different taxonomic levels in faeces from STC mice treated with As‐IV. The microbiome profile alterations among the five mouse groups were compared at the levels of phylum (A), order (C) and genus (E). Relative abundances of Firmicutes (B), Lactobacillales (D), Lactobacillus (F) and Lactobacillus reuteri (G) in faecal samples collected from STC mice treated with As‐IV. As‐IV, astragaloside IV; STC, slow transit constipation; LD, low dosage; MD, median dosage; HD, high dosage; *P < .05, **P < .01 (comparison with control group); ^#^ *P* < .05, ^##^ *P* < .01 (comparison with STC group)

We analysed the alterations of gut microbiota profiles induced by As‐IV treatment in STC mice at different taxonomic levels. At the phylum level, the microbiomes identified in this study were mainly composed of Firmicutes, Bacteroidetes, Verrucomicrobia, Proteobacteria and Actinobacteria (Figure 4A). Among these, Firmicutes made up the largest proportion and its relative abundance was significantly reduced in the STC group compared with that in the control, but was then recovered by As‐IV treatment (Figure 4B). At the order level, the microbiota community mainly consisted of Bacteroidales, Lactobacillales, Clostridiales and Verrucomicrobiales, which covered over 70% of all microbial species (Figure 4C). Compared with the control group, the relative abundance of Lactobacillales was remarkably reduced in the STC group and was also recovered by As‐IV (Figure 4D). At the genus level, we found that Lactobacillus and Akkermansia shared the highest relative abundance (Figure 4E). The relative abundance of Lactobacillus was also decreased in the STC group compared with that in the control group, but was elevated by As‐IV treatment (Figure 4F). We also observed that the abundance of Lactobacillus reuteri in the STC group was much lower than that in the control group, but was also recovered by As‐IV treatment (Figure 4G). These results indicate that changes in the gut microbiota community structure during STC pathogenesis can potentially be reversed by As‐IV treatment.

### 3.4 Astragaloside IV increased butyric and valeric acid but reduced isovaleric acid content in STC mouse stools

**Figure 5:**
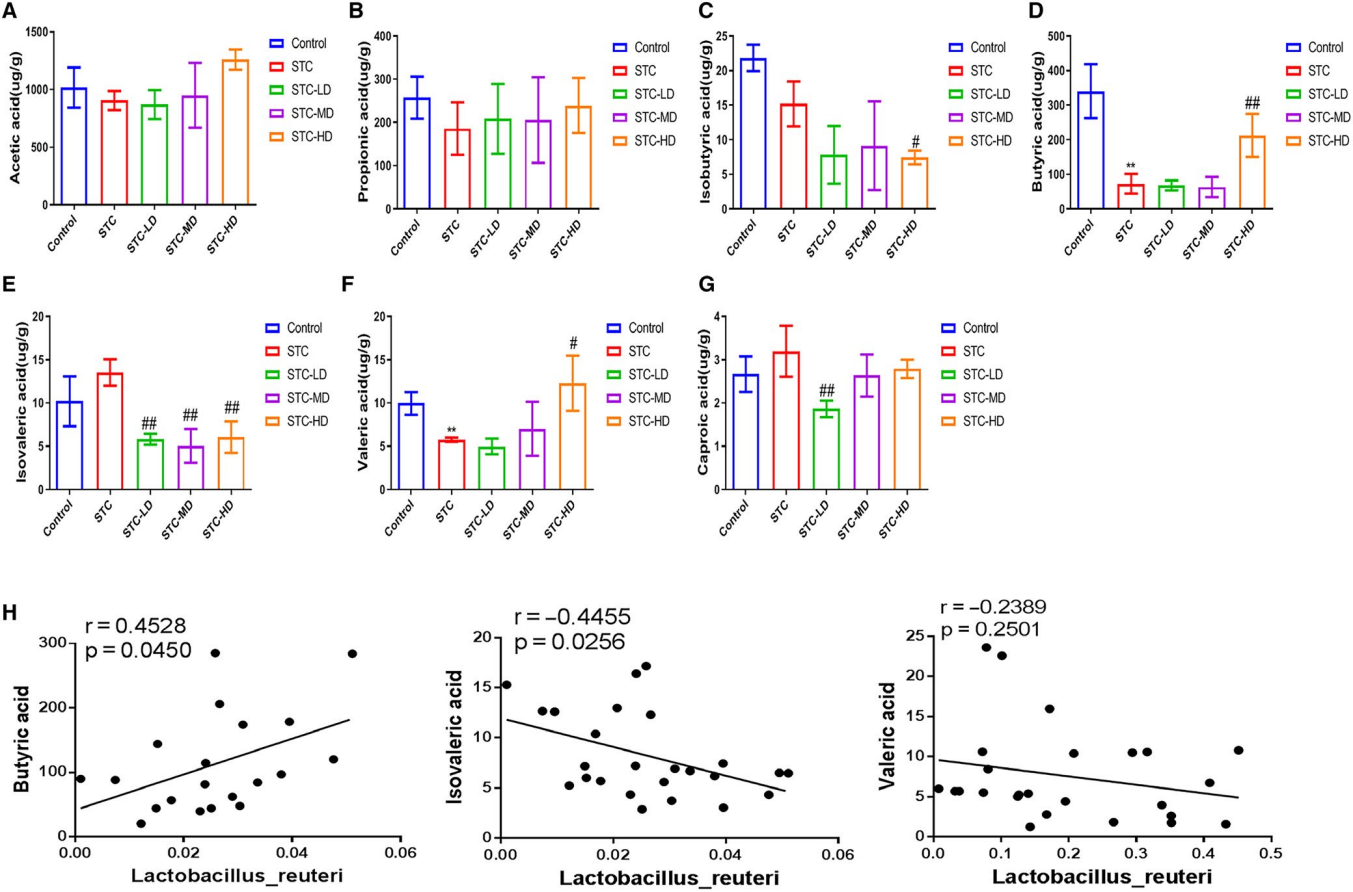
Regulation of short‐chain fatty acid generation by astragaloside IV in STC mice. A–G, The levels of major short‐chain fatty acids in the stools from STC mice treated with As‐IV. Gas chromatography‐mass spectrometry (GC/MS) was used to determine the levels of acetic acid (A), propionic acid (B), isobutyric acid (C), butyric acid (D), isovaleric acid (E), valeric acid (F) and caproic acid (G) in stools from the control, STC, STC‐LD, STC‐MD and STC‐HD groups. H, The correlations of *Lactobacillus reuteri* abundance in mouse gut microbiome with the levels of butyric acid (left), isovaleric acid (middle) and valeric acid (right) in mouse stools. Significances of the correlations were analysed using the Pearson method. As‐IV, astragaloside IV; STC, slow transit constipation; LD, low dosage; MD, median dosage; HD, high dosage; **P < .01 (comparison with control group); ^#^ *P* < .05, ^##^ *P* < .01 (comparison with STC group)

To study the possible influence of As‐IV on SCFA generation, we determined the levels of the major SCFA members in the stool samples of STC mice after As‐IV treatment, including acetic acid (Figure 5A), propionic acid (Figure 5B), isobutyric acid (Figure 5C), butyric acid (Figure 5D), isovaleric acid (Figure 5E), valeric acid (Figure 5F) and caproic acid (Figure 5G). We found that the levels of butyric acid and valeric acid in stools from the STC group were significantly lower than in the control group, but were greatly elevated by treatment with high‐dose As‐IV (Figure 5D,F. In contrast, the level of isovaleric acid in the STC group was slightly higher than that in the control group, but was significantly down‐regulated by As‐IV under different dosages (Figure 5E). We showed that the relative abundance of *Lactobacillus reuteri* in these mouse groups was positively correlated with the level of butyric acid, but negatively correlated with the level of isovaleric acid in mouse stools (Figure 5H). No significant correlation was observed between *Lactobacillus reuteri* abundance and valeric acid content (Figure 5H). These results suggest that As‐IV may modulate the abundance of *Lactobacillus reuteri* and butyric acid generation to alleviate the symptoms of constipation.

### 3.5 Butyrate improved STC mouse defecation and intestinal mobility by regulating AKT (protein kinase B)‐NF‐κB (nuclear factor‐kappaB) signalling

**Figure 6:**
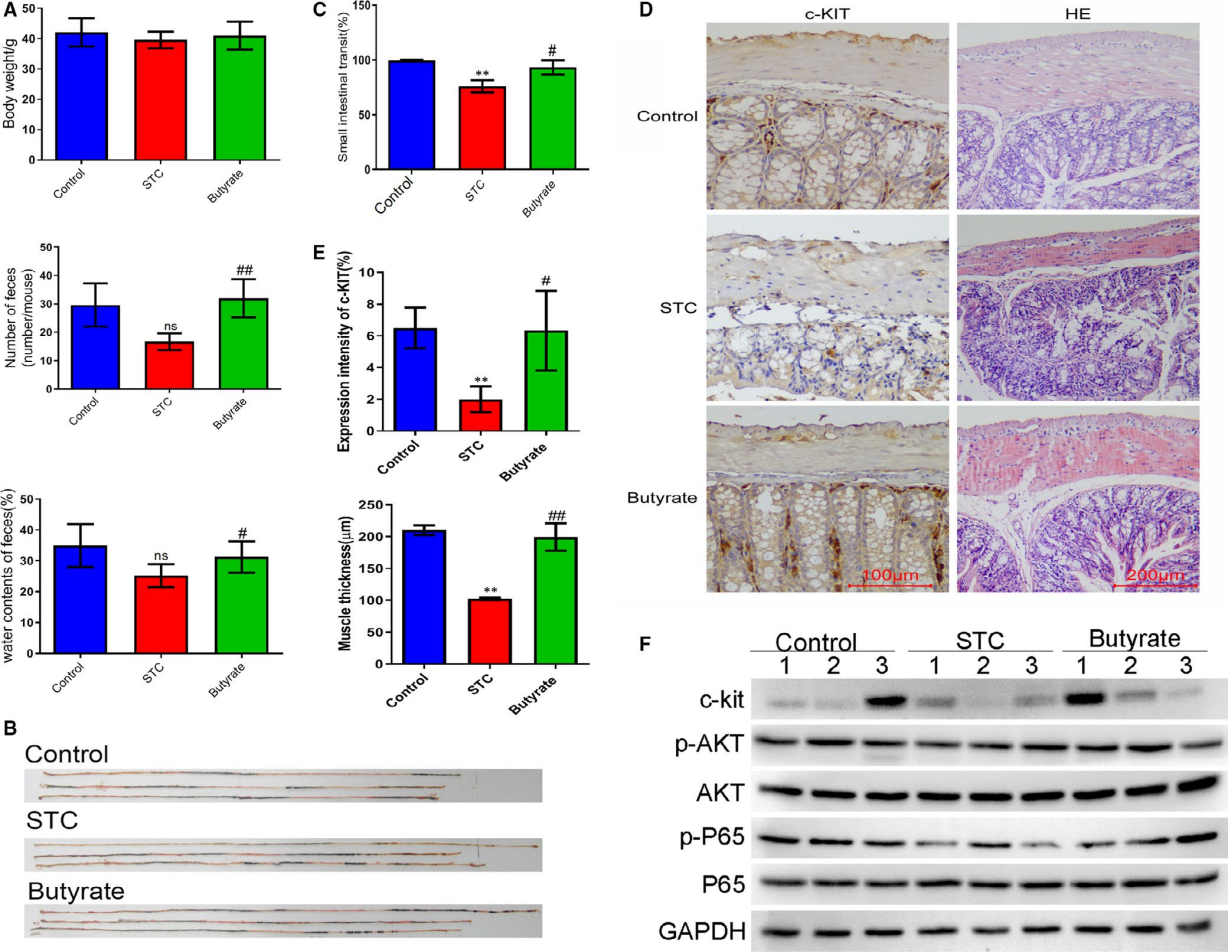
Recovery of defecation and intestinal transit in STC mice by butyrate. A, The bodyweight, faeces number and faecal water content of STC mice treated with sodium butyrate. Twenty‐four mice were divided into a control group, STC group and STC + butyrate group. B, C, Effects of sodium butyrate treatment on small intestinal transit of STC mice. The intestinal propelling movement of carbon ink (B) was performed to analyse mouse small intestinal transit, followed by statistical analysis (C). D, The c‐Kit expression and pathogenic alterations in the colonic tissues of STC mice treated with sodium butyrate. E, Quantitation of c‐Kit expression and muscle thickness in the colonic tissues of STC mice following sodium butyrate treatment. F, Relative abundance of c‐Kit, P65, p‐P65, AKT and p‐AKT proteins in the colons of sodium butyrate‐treated STC mice. GAPDH was used as the internal standard. STC, slow transit constipation; HE, haematoxylin and eosin; p‐P65, phosphorylated P65; AKT, protein kinase B; p‐AKT, phosphorylated AKT; GAPDH, glyceraldehyde‐3‐phosphate dehydrogenase; *P < .05, **P < .01 (comparison with control group); ^#^ *P* < .05, ^##^ *P* < .01 (comparison with STC group). ns, not significant

To investigate the role of butyric acid (butyrate) in regulating the pathogenesis of STC, we treated mice in the STC group with sodium butyrate. We showed that the bodyweights of mice in the STC group were not altered by butyrate treatment, but the number of faeces and the faecal water content of the STC group were remarkably increased by butyrate treatment (Figure 6A). As evaluated by the intestinal propelling movement of carbon ink, we also observed that the small intestinal transit of the STC group, which was greatly repressed in comparison with that in the control group, was effectively recovered by butyrate treatment (Figure 6B,C). Butyrate treatment significantly enhanced c‐Kit expression in colonic tissues, and greatly alleviated mucosal goblet cell loss, myenteric plexus dysregulation, lymphocyte infiltration, muscle thickness reduction and other constipation‐related colonic lesions in the STC group (Figure 6D,E). Western blotting showed that the abundances of c‐Kit, phosphorylated P65 (p‐P65), and phosphorylated AKT (p‐AKT) proteins in colonic tissues in the STC group were all markedly lower than those in the control group, but were significantly elevated by sodium butyrate treatment (Figure 6F). These results clearly show the defecation‐ and intestinal mobility‐promoting role of butyrate in STC mouse colons.

### 3.6 Butyrate promoted mouse ICC proliferation by activating AKT/NF‐κB signalling

**Figure 7:**
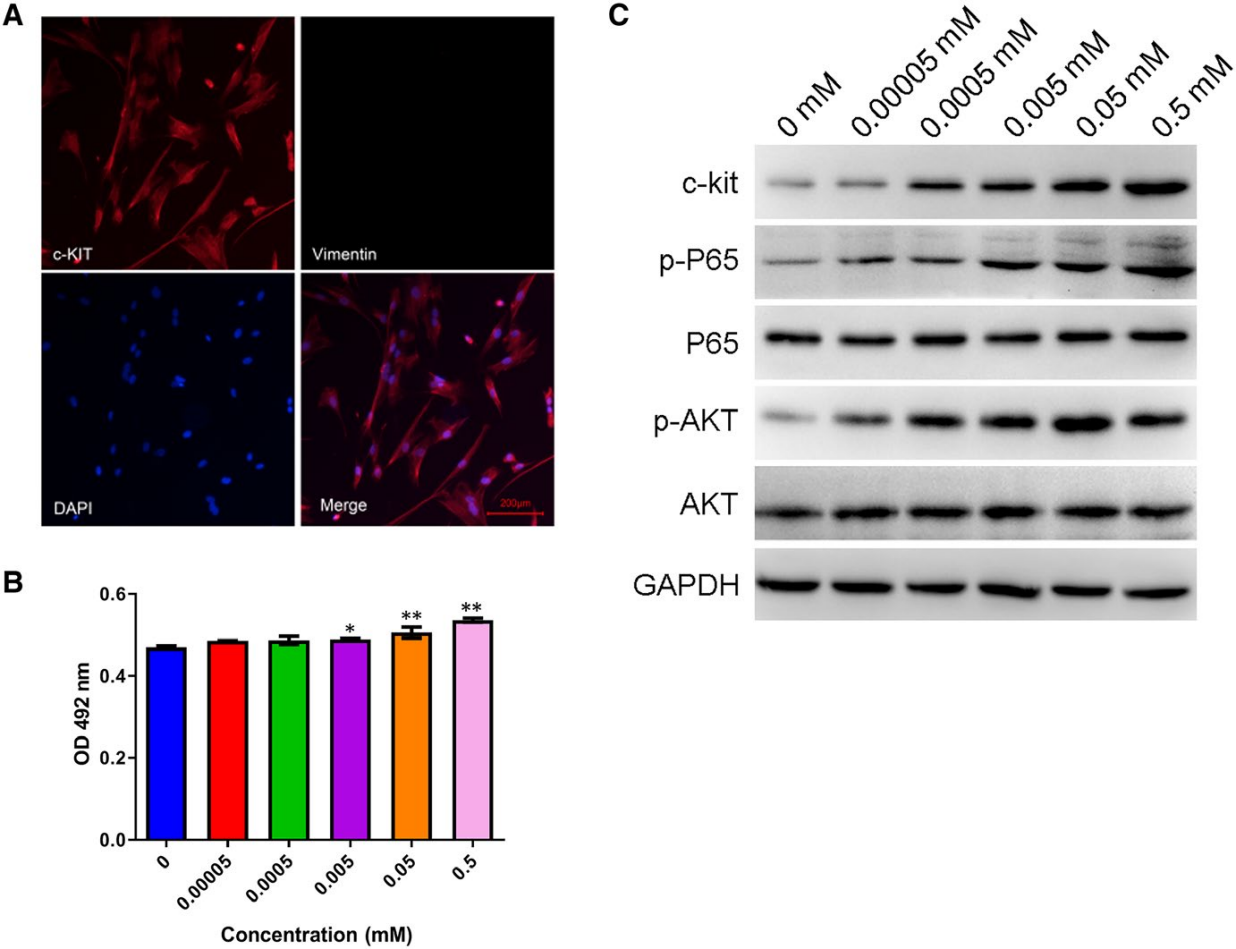
Enhancement of ICC proliferation by butyrate through activation of AKT/NF‐κB signalling. A, The expression of c‐Kit and vimentin proteins in ICC cells isolated from mouse colon tissues. Protein expression was measured by the immunofluorescence method using antibodies targeting c‐Kit and vimentin proteins. Scale bar = 200 μm. B, Influence of sodium butyrate treatment on mouse ICC cell proliferation. The proliferation rate of mouse ICC cells was evaluated by the MTS method after treatment with sodium butyrate at the designated concentration for 4 h. C, Relative levels of c‐kit, P65, p‐P65, AKT and p‐AKT proteins in mouse ICC cells treated with sodium butyrate. Protein levels were detected by Western blotting, and GAPDH was tested as an internal standard. MTS, 3‐(4,5‐Dimethylthiazol‐2‐yl)‐5‐(3‐Carboxymethoxyphenyl)‐2‐(4‐Sulfophenyl)‐2H‐terazolium; p‐P65, phosphorylated P65; AKT, protein kinase B; p‐AKT, phosphorylated AKT; GAPDH, glyceraldehyde‐3‐phosphate dehydrogenase; ICC, interstitial cells of Cajal; *P < .05, **P < .01

For further insight into the molecular mechanisms behind the regulation of defecation and colon mobility by butyrate, we isolated ICCs from mouse colonic tissues, as described in the Material and Methods section of this paper. Through immunofluorescence, we detected significant expression of c‐Kit proteins, but no vimentin proteins in isolated mouse ICC cells, which confirmed the identity of the isolated ICC cells (Figure 7A). The ICC cells were then treated with 0, 0.00005, 0.0005, 0.005, 0.05 and 0.5 mmol/L sodium butyrate, which resulted in increases in ICC cell proliferation in a butyrate concentration‐dependent manner (Figure 7B). Significant enhancement of ICC cell proliferation was obtained by treatment with 0.05 or 0.5 mmol/L sodium butyrate, as shown by the 3‐(4,5‐Dimethylthiazol‐2‐yl)‐5‐(3‐Carboxymethoxyphenyl)‐2‐(4‐Sulfophenyl)‐2H‐terazolium (MTS) cell viability assay (Figure 7B). Moreover, we found that the c‐Kit, p‐P65 and p‐AKT protein levels in the isolated mouse ICC cells were also remarkably elevated by sodium butyrate treatment in a concentration‐dependent manner (Figure 7C). These results show that sodium butyrate promoted the proliferation of mouse ICC cells by activating the AKT/NF‐κB signalling pathway.

## 4 DISCUSSION

The interactions of the gut microbiome and host microbes, and their SCFA fermentation products, have been shown to be closely associated with various gastrointestinal and metabolic disorders.^10^, ^11^, ^12^, ^16^, ^23^, ^24^ Modulation of the gut microbiome and SCFA metabolism has emerged as a promising strategy for new drug development. However, little is known about the pathogenic role of the gut microbiome in STC, which has greatly hampered the development of anti‐constipation treatments targeting the microbiota structure and SCFA metabolism. In the present study, we demonstrated for the first time that As‐IV effectively promoted defecation and colonic mobility and also alleviated constipation‐associated colonic lesions in a loperamide‐induced mouse STC model, mediated by an increase in ICCs. Significant alterations in the gut microbiota community structures and SCFA content were characterized in the STC model, which were recovered following As‐IV treatment. We observed correlative changes of *Lactobacillus reuteri* with the levels of butyric acid and isovaleric acid in mouse stools. Finally, the effects of butyric acid on colonic mobility and ICC cell proliferation were confirmed and were shown to be associated with STC development and the inhibition of STC symptoms following treatment with As‐IV. These results provide novel insights into the molecular pathogenesis of STC and lay the foundation for developing new drugs for STC and other gastrointestinal diseases.

*AM* is an important herbal plant that is widely used in traditional Chinese medicine and has a long history of medicinal usage.^25^, ^26^ *AM* and its extracts are commonly used for the clinical management of various human disorders, such as vascular disorders,^26^ obesity,^27^ diabetic nephropathy,^28^ breast cancer^29^ and colon cancer.^24^ Recent pharmaceutical analysis has found that the therapeutic effects of *AM* may be attributable to the major bioactive component As‐IV.^15^, ^30^ However, the use of As‐IV in the treatment of constipation has not been previously studied. In this study, we showed that the small intestinal transit of STC mice was significantly improved by As‐IV, which improved defecation and promoted the remission of major colonic lesions associated with STC. These findings identify As‐IV as a new candidate drug for the treatment of constipation. In addition to As‐IV, previous research showed that *AM* contains other bioactive constituents, such as fatty acid esters and galactosyl acylglycerols,^31^ which may also exert inhibitory effects on STC and constipation‐associated diseases. Based on the results of this animal study, the therapeutic effects of As‐IV and other *AM* components warrant further investigation in large‐scale clinical studies.

Abnormal alterations in gut microbiota communities are closely associated with various pathogenic conditions.^9^, ^11^ Modulation of the structure and diversity of the gut microbiota community are regarded as a promising new approach for preventing and treating disease. For example, omega‐3 polyunsaturated fatty acids were recently reported to critically modulate the development of the gut microbiota, which may be involved in neurodevelopment in adolescence and adulthood.^32^ A number of gut microbiota regulators have recently been examined for their health benefits, such as primary‐response gene 88 (MyD88)^33^ and taurine.^34^ We noted significant recovery of the gut microbiota community in the colonic tissues of mice with STC following the administration of As‐IV, including *Lactobacillus reuteri* (DM 17938), which is reported to be beneficial in functional constipation because of its effects on the frequency of bowel movements.^35^ The results of this study show that As‐IV is an effective new gut microbiome modulator and also suggest that herbal plants could be a reliable source for screening gut microbiome regulators with potent therapeutic effects. Microbiota can also regulate intestinal mucosal function and immune response by liberating SCFAs, such as the abundant bioactive butyrate, from dietary fibres.^9^ The pathogenesis of functional constipation is reported to be associated with the altered concentrations of butyric acids.^36^ In this study, we detected a significant increase in butyric acid in STC mouse stools following As‐IV treatment and also proved that sodium butyrate could promote defecation and colonic mobility in STC mice. These results validate the anti‐constipation roles of As‐IV and butyrate, suggesting the potential of As‐IV to be used as a therapeutic agent for STC.

The loss of ICC cells is implicated in the pathogenesis of multiple human disorders, such as chronic unexplained nausea and vomiting,^37^ small bowel obstruction,^38^ gastric dysrhythmia in streptozotocin‐induced diabetes^39^ and STC.^40^ ICCs play an essential role in regulating the symphony of gut motility by acting as the pacemakers of the gastrointestinal muscles.^41^ The maintenance of ICC phenotypes and functions in the gastrointestinal system greatly depends on signalling cascades, which are mediated by the expression of tyrosine kinase receptor c‐Kit protein on the cell surface.^42^ Previous reports showed that c‐Kit, the gastrointestinal proto‐oncogene, is a specific marker for ICC.^43^ In the present study, we demonstrated that the loss of ICC cells and decrease in c‐Kit expression during the pathogenesis of STC were effectively suppressed by As‐IV and sodium butyrate. We directly showed the ICC proliferation‐enhancing effects of sodium butyrate using ICC cells isolated from STC mice. In addition, sodium butyrate could promote the expression of p‐P65/P65 and pAKT/AKT in ICC cells and colon tissues of STC mice. It was reported that NF‐κB and AKT signalling are well‐known regulators of cell proliferation,^44^, ^45^ which are also involved in modulating the pacemaker activity of ICC cells.^46^, ^47^ These findings revealed that butyrate promoted intestinal motility by activating the NF‐κB and AKT signalling in ICCs. This highlights the roles of ICC cells as targets in the treatment of STC and supports the role of As‐IV in inhibiting STC pathogenesis by regulating gut microbiota and promoting SCFA generation.

This study showed that As‐IV effectively promoted intestinal transit in mice with loperamide‐induced STC by inhibiting the loss of ICCs, mediating the regulation of the gut microbiota community structure and enhancing butyric acid generation. The results of this study provide novel insights into the role of the gut microbiome in the pathogenesis of STC and indicate the potential of As‐IV as a therapeutic agent in STC.

## CONFLICT OF INTEREST

The authors declare that they have no conflict of interest.

## AUTHOR CONTRIBUTIONS

**Qiulan He:** Data curation (equal); formal analysis (equal); investigation (equal); methodology (equal); visualization (equal); writing‐original draft (lead). **Changpeng Han:** Data curation (equal); formal analysis (lead); funding acquisition (equal); investigation (equal); software (equal); writing‐original draft (equal). **Liang Huang:** Data curation (equal); investigation (equal); methodology (lead); software (equal); writing‐original draft (equal). **Haojie Yang:** Data curation (equal); investigation (equal); methodology (equal); validation (equal). **Jiancong Hu:** Data curation (equal); investigation (equal); software (equal); validation (equal). **Huaxian Chen:** Data curation (equal); formal analysis (equal); investigation (equal). **Ruoxu Dou:** Investigation (equal); validation (equal); visualization (equal). **Donglin Ren:** Conceptualization (equal); project administration (equal); supervision (equal); writing‐review and editing (equal). **Hongcheng Lin:** Conceptualization (lead); funding acquisition (equal); project administration (equal); resources (lead); writing‐review and editing (equal).

## ETHICAL APPROVAL

All animal experiments in this study were approved by the Animal Care Review Committee of the Sun Yat‐sen University and carried out strictly according to the Guidelines for the Care and Use of Laboratory Animals (National Institutes of Health, USA).

## Data Availability

The datasets generated during and/or analysed during the current study are available from the corresponding author on reasonable request.
